# The Peptide ERα17p Is a GPER Inverse Agonist that Exerts Antiproliferative Effects in Breast Cancer Cells

**DOI:** 10.3390/cells8060590

**Published:** 2019-06-14

**Authors:** Rosamaria Lappano, Christophe Mallet, Bruno Rizzuti, Fedora Grande, Giulia Raffaella Galli, Cillian Byrne, Isabelle Broutin, Ludivine Boudieu, Alain Eschalier, Yves Jacquot, Marcello Maggiolini

**Affiliations:** 1 Department of Pharmacy, Health and Nutritional Sciences, University of Calabria, 87036 Rende, Italy; rosamaria.lappano@unical.it (R.L.); fedora.grande@unical.it (F.G.); giulia.r.galli@gmail.com (G.R.G.); 2 NEURO-DOL Basics & Clinical Pharmacology of Pain, INSERM, CHU, Université Clermont Auvergne, F-63000 Clermont-Ferrand, France; christophe.mallet@uca.fr (C.M.); ludivine.boudieu@uca.fr (L.B.); alain.eschalier@uca.fr (A.E.); 3 ANALGESIA Institute, Université Clermont Auvergne, F-63000 Clermont-Ferrand, France; 4 CNR-NANOTEC, Licryl-UOS Cosenza and CEMIF.Cal, Department of Physics, University of Calabria, 87036 Rende, Italy; bruno.rizzuti@fis.unical.it; 5 Laboratoire des Biomolécules (LBM), CNRS-UMR 7203, Sorbonne University, Ecole Normale Supérieure, 75252 Paris Cedex 05, France; cillian.byrne@upmc.fr; 6 Cibles Thérapeutiques et Conception de Médicaments (CiTCoM), CNRS–UMR 8038, Faculté des Sciences Pharmaceutiques et Biologiques, Université Paris Descartes, 75270 Paris Cedex 06, France; isabelle.broutin@parisdescartes.fr

**Keywords:** peptide, anti-proliferation, inverse agonist, desensitizer, GPER, SkBr3, breast cancer

## Abstract

The inhibition of the G protein-coupled estrogen receptor (GPER) offers promising perspectives for the treatment of breast tumors. A peptide corresponding to part of the hinge region/AF2 domain of the human estrogen receptor α (ERα17p, residues 295–311) exerts anti-proliferative effects in various breast cancer cells including those used as triple negative breast cancer (TNBC) models. As preliminary investigations have evoked a role for the GPER in the mechanism of action of this peptide, we focused our studies on this protein using SkBr3 breast cancer cells, which are ideal for GPER evaluation. ERα17p inhibits cell growth by targeting membrane signaling. Identified as a GPER inverse agonist, it co-localizes with GPER and induces the proteasome-dependent downregulation of GPER. It also decreases the level of pEGFR (phosphorylation of epidermal growth factor receptor), pERK1/2 (phosphorylation of extracellular signal-regulated kinase), and c-fos. ERα17p is rapidly distributed in mice after intra-peritoneal injection and is found primarily in the mammary glands. The N-terminal PLMI motif, which presents analogies with the GPER antagonist PBX1, reproduces the effect of the whole ERα17p. Thus, this motif seems to direct the action of the entire peptide, as highlighted by docking and molecular dynamics studies. Consequently, the tetrapeptide PLMI, which can be claimed as the first peptidic GPER disruptor, could open new avenues for specific GPER modulators.

## 1. Introduction

The 66 kDa human estrogen receptor α (ERα) is a transcription factor belonging to the superfamily of steroid hormone receptors [1]. This ubiquitous protein is widely distributed in the uterus, ovaries, breast tissue, and bones, as well as in the central nervous and cardiovascular systems. At the cellular scale, it is located in the nucleus, within membranes including the caveolae [2] and the mitochondria [3] where it displays distinct effects.

In addition to the palmitoylated ERα [4], two truncated ERα isoforms (i.e., 36 kDa [5] and 46 kDa [6]) have been identified at the cell membrane. An additional estrogen-interacting hepta-transmembrane G protein-coupled estrogen receptor 1 (GPER1 or GPER), also called G protein-coupled receptor 30 (GPR30), has been described [7]. GPER participates in the action of estrogens through growth factor receptors including the epidermal growth factor receptor (EGFR) to activate mitogen-activated protein kinases (MAPK) such as the extracellular signal-regulated kinase (ERK1/2) [8]. Thus, GPER is a real target for the treatment of breast tumors [9].

In the ERα, the hinge region, delimited by the residues 263 and 302, spatially links the C and E/F domains and participates, as such, in the control of transcription [10]. Besides the K^303^NSLALSLT^311^ N-terminal sequence of the ERα E domain, amino acids located at the C-terminal region of the D domain (sequence: P^293^SPLMIKRSK^303^) are particularly subjected to post-translational modifications. The 295–311 sequence is prone to acetylation [11,12,13], phosphorylation [14,15], methylation [16], ubiquitination [12,17], and SUMOylation [18], suggesting that this region of the receptor is important for transcription. Accordingly, the 295–311 deleted protein (ERαΔ295-311) is constitutively active [19]. As such, the sequence 302–339 is considered as a part of the ERα autonomous transactivation function AF2a [20]. In the same context, the motif K^299^RSKK^303^, which corresponds to the third nuclear receptor localization signal [21], is sensitive to proteolysis [22,23]. The close proximity of this cationic motif to the cysteine 447 palmitoylation site (~15 Å) and its ability to associate with anionic phospholipids suggests that it could participate in the stabilization of the protein at the membrane [24]. The K303R mutation, which seems to participate in tamoxifen resistance, has been found in invasive breast tumors, highlighting the importance of this part of the protein in malignancy onset [25,26]. In the context of the whole receptor, the 295–311 sequence is located at the surface of the ERα and belongs to a larger flexible helix (residues 295–330) partially folded in an extended left-handed polyproline II (PPII) conformation [27]. This observation implies that some interactions with protein partners could occur [28]. Apart from its association with Ca^2+^-calmodulin [19,29,30] and DNA-bond c-jun [31], the 395–311 part of the ERα interacts intramolecularly with the sole ERα type II β turn of the ligand-binding domain (LBD, sequence: V^364^PGF^367^ [27]), which can associate with the proline-rich nuclear receptor co-activator (PNRC) [32] and possibly with the protein FKBP52 [33,34]. In the light of the aforementioned observations, this part of the ERα, albeit small in size, poses more questions than it provides answers. Consequently, the short D domain has garnered considerable interest during the last decade.

We have thus synthesized the human ERα-derived peptide PLMIKRSKKNSLALSLT (ERα17p, residues 295 to 311) and tested its effects in different experimental conditions and in various ERα-positive (ERα+) and -negative (ERα−) breast cancer cell lines. Under physiological conditions, it elicits apoptosis and necrosis independently from the ERα status [35]. After intraperitoneal injections at low concentration (1.5 mg/kg, three times a week for four weeks), this peptide decreases by ~50% the size of ERα-related human breast tumors (MDA-MB-231 breast cancer cells) xenografted in nude mice [35]. In the light of these substantial results, the peptide ERα17p could be a putative anticancer drug active on triple negative breast tumors (TNBC) for which no specific treatment currently exists.

We have attempted to probe the mechanism of action of ERα17p in physiological conditions (i.e., in complete serum) by focusing on membrane-initiated signaling pathways. We show that the peptide co-localizes with the GPER in ERα-negative SKBR3 breast cancer cells. Identified as an inverse agonist, it decreases the basal activity of GPER and triggers anti-proliferative activity. These effects are concurrent with a proteasome-dependent GPER downregulation, a decreased phosphorylation of EGFR and ERK1/2, and a decrease of the level of c-fos. It also targets the ovaries, the uterus and, seemingly, the mammary glands. Following docking and molecular dynamics studies, the N-terminal PLMI tetrapeptide motif, which presents structural similarities with the GPER ligand PBX1 [36] and which most likely supports the action of ERα17p, is predicted to interact within the GPER ligand-binding site.

## 2. Materials and Methods

### 2.1. Peptide Synthesis

All chemicals were purchased from Sigma Aldrich (Saint-Quentin-Fallavier, France). The peptides were manually synthesized via Fmoc solid phase peptide synthesis (SPPS) using a preloaded Fmoc-Thr-Novasyn TGA resin (Merck, Fontenay sous Bois, France), as previously described [24,37]. Peptide cleavage and side-chain deprotection was carried out prior to lyophilization, purification, and characterization. The crude products were purified by semi-preparative reverse-phase high performance liquid chromatography RP-HPLC (Waters, Saint-Quentin en Yveline, France) using a Waters 600 pump and controller and a 2487 UV–Vis detector (λ = 220 nm, flow rate = 5 mL/min). Solvents used for elution: A (milliQ water with 0.1% of trifluoroacetic acid (TFA)) and B (acetonitrile:milliQ water (90:10) with 0.1% TFA). Analytical RP-HPLC conditions: λ = 220 nm, flow rate = 1 mL/min. The purified peptides were then characterized by matrix assisted laser desorption ionization time of flight (MALDI-TOF) mass spectrometry using a 4700 Proteomic Analyzer (Applied Biosystems, Foster City, CA, USA). α cyano-4-hydroxycinnamic acid (HCCA) was used as a matrix. Characterization data of the peptide ERα17p (PLMIKRSKKNSLALSLT) as well as of its fragments (PLMI) are reported elsewhere [24,37]. The scrambled peptide (KLSKNKRLMTISPLSLA) was purchased from the Plateforme d’Ingénierie des Protéines (Christophe Piesse, Institut de Biologie Paris-Seine, IBPS, FR 3631, Paris, France).

N-terminal ERα17p labeling was carried out by using 5(6)-carboxyfluorescein (fluorescein-Ahx-ERα17p-COOH, where Ahx corresponds to aminohexanoic acid), following the standard Fmoc peptide synthesis protocol [24,37]. A SymetriPrep C_8_ column (7.8 mm × 300 mm, 7 μm particle size, 300 Å pore size, Waters, Saint-Quentin en Yveline, France) and appropriate eluent gradient (30% to 60% of solvent B over 20 min) were used for semi-preparative RP-HPLC. R_t_ = 10.4 min. Analytical RP-HPLC was carried out using an ACE 5 C_18_ 300 Å. RP-HPLC conditions: 5% to 60% of solvent B over 20 min; R_t_ = 17.13 min. Calculated isotopic *m*/*z* = 2369.29 (found: 2369.21).

The sequence H_2_N-ERα17p-Pra-COOH, where Pra corresponds to propargyl glycine, was obtained by standard Fmoc peptide synthesis [24,37]. The Pra was used for the synthesis of the “click” Cy5-labeled version of ERα17p. Briefly, the purified peptide H_2_N-ERα17p-Pra-COOH (3 mg, 1.16 µmol) and Cy5 azide (1 mg, 0.97 µmol) were dissolved in water (1 mL). To this was added, with stirring, 1.2 mg of CuSO_4_.5H_2_O (4.83 µmol) in 100 µL water:DMF (95:5). Sodium ascorbate (4.8 mg, 24.1 µmol) was then added to this solution. The mixture was stirred for 30 min and purified directly by RP-HPLC. The recovered fractions were freeze-dried to yield a deep red powder (1.5 mg, yield = 33%). An Xbridge RP C_18_ column (30 × 100 mm) was used for purification. Semi-preparative RP-HPLC conditions: 20–40% of solvent B over 10 min. R_t_ = 7.6 min. Analytical RP-HPLC was carried using an Agilent technologies Ultimate 3000 pump, autosampler and RS UV–Vis variable wavelength detector with a Higgins Analytical Proto 300 C18 column (4.6 × 100 mm). Analytical RP-HPLC conditions: 15–80% of solvent B over 10 min. R_t_ = 6.28 min. Calculated isotopic *m*/*z* = 2827.44 (found: 2826.31).

### 2.2. Fluorescence Spectroscopy

The recombinant Grb2 protein was obtained and purified following a previously published protocol [38]. The interaction of ERα17p with Grb2 SH3 domains was estimated using a fluorescence-based titration assay, which was performed at 18 °C in a 1 cm pathlength cell with stirring using a Jasco FP-6200 spectrofluorimeter (Jasco, Essex, United Kingdom). Excitation and emission wavelengths were fixed at 280 and 350 nm, respectively. A Grb2 concentration of 1 μM in 50 mM Tris buffer adjusted to pH 8.0 was initially used. Fluorescence changes were recorded upon the addition of 5 μL of a peptide solution at 10^−3^ M. The experimental curve was analyzed with the software Prism™ (version 5.0a, GraphPad Software, San Diego, California, USA). The experiment was performed twice.

### 2.3. Cell Growth Assays

17β-Estradiol (E_2_) and MG-132 were purchased from Sigma-Aldrich (Milan, Italy) and solubilized in ethanol and DMSO, respectively. G-1 and G-36 were bought from Tocris Bioscience (Bristol, UK) and dissolved in DMSO. SkBr3 breast cancer cells were obtained by ATCC and used less than 6 months after resuscitation. The cells were maintained in RPMI 1640 without phenol red but supplemented with 5% fetal bovine serum (FBS) and 100 mg/mL penicillin/streptomycin (Life Technologies, Milan, Italy). Cells were grown in a 37 °C incubator with 5% CO_2_.

Cells were seeded in 24-well plates in regular growth medium. After cells attached, they were incubated in medium containing 2.5% charcoal-stripped fetal bovine serum (FBS) and treated for 72 h either in the presence or absence of the tested molecules. Treatments were renewed every day. Cells were counted on day 4 using an automated cell counter (Life Technologies, Milan, Italy), following the manufacturer’s recommendations.

### 2.4. TUNEL Experiments

Cell apoptosis was determined by TdT-mediated dUTP Nick-End Labeling (TUNEL) assay [39] conducted using a DeadEnd Fluorometric TUNEL System (Promega, Milan, Italy) and performed according to the manufacturer’s instructions. Briefly, cells were treated for 72 h under various conditions (see figure legends), then were fixed in freshly prepared 4% paraformaldehyde solution in PBS (pH 7.4) for 25 min at 4 °C. After fixation, they were permeabilized in 0.2% Triton X-100 solution in PBS for 5 min. After washing twice with washing buffer for 5 min, the cells were covered with equilibration buffer at room temperature for 5 to 10 min. The labeling reaction was performed using terminal deoxynucleotidyl transferase end-labeling TdT and fluorescein-dUTP cocktail for each sample and incubated for 1 h at 37 °C, where TdT catalyzes the binding of fluorescein-dUTP to free 3′OH ends of the nicked DNA. After rinsing, the cells were washed with 2× saline-sodium citrate (SSC) solution buffer and subsequently incubated with 4′,6-diamidino-2-phenylindole (DAPI; Sigma-Aldrich, Milan, Italy) to stain nuclei and then analyzed using the Cytation 3 Cell Imaging Multimode Reader (BioTek, Winooski, VT, USA).

### 2.5. Fluorescence Microscopy

Cells were seeded in Lab-Tek II chamber slides at a density of 1 × 10^5^ per well and incubated for 24 h in the maintenance medium. Cells were then treated as specified (see the legends of the figures), fixed in 4% paraformaldehyde, permeabilized with 0.1% TWEEN three times for 5 min and were then blocked for 30 min at room temperature with PBS containing 10% normal donkey serum (Santa Cruz Biotechnology, DBA, Milan, Italy), 0.1% Triton X-100, and 0.05% TWEEN (3 × 5 min). Thereafter, the cells were incubated overnight at 4 °C with a primary antibody against GPER (TA35133, 1:250, purchased from Origene, DBA, Milan, Italy) in PBS containing 0.05% TWEEN. After incubation, the slides were extensively washed with PBS and incubated with Alexa Fluor^TM^ 594 goat anti-rabbit IgG (H + L) (1:250, purchased from Life Technologies, Milan, Italy). The slides were imaged on the Cytation 3 Cell Imaging Multimode reader (BioTek, Winooski, VT, USA).

### 2.6. Immunoblotting

Cells were grown in 10-cm dishes exposed to treatments, and then lysed in 500 μL of 50 mmol/L NaCl, 1.5 mmol/L MgCl_2_, 1 mmol/L EGTA, 10% glycerol, 1% Triton X-100, 1% sodium dodecyl sulfate (SDS), and a mixture of protease inhibitors containing 1 mmol/L aprotinin, 20 mmol/L phenylmethylsulfonyl fluoride and 200 mmol/L sodium orthovanadate. Equal amounts of whole protein extracts were resolved on a 10% SDS-polyacrylamide gel and transferred to a nitrocellulose membrane (Amersham Biosciences, GE Healthcare, Milan, Italy), which were probed with primary antibodies against GPER (TA 35133, OriGene, Herford, Germany), pEGFR Tyr-1173 (sc-12351), EGFR (1005), phosphorylated ERK1/2 (E-4), ERK2 (C-14), c-fos (E-8), and β-actin (C2) (Santa Cruz Biotechnology, DBA, Milan, Italy) and then revealed using the ECL™ system from GE Healthcare (Milan, Italy).

### 2.7. Gene Expression Studies

Total RNA was extracted, and cDNA was synthesized by reverse transcription as previously described [40]. The expression of selected genes was quantified by real-time PCR using a Quant Studio7 Flex Real-Time PCR System platform (Applied Biosystems Inc, Milan, Italy). Gene-specific primers were designed using Primer Express version 2.0 software (Applied Biosystems Inc, Milan, Italy). For GPER and actin, whose genes were used as controls to obtain normalized values, the primers were 5’-ACACACCTGGGTGGACACAA-3’ (GPER forward) and 5’-GGAGCCAGAAGCCACATCTG-3’ (GPER reverse) as well as 5′-AAGCCACCCCACTTCTCTCTAA-3′ (actin forward) and 5′-CACCTCCCCTGTGTGGACTT-3′ (actin reverse), respectively. The assays were performed in triplicate and the results were normalized for actin expression and then calculated as fold induction of RNA expression.

### 2.8. In Vivo and Ex Vivo Fluorescence Imaging

Female mice RjOrl:SWISS (30 g, Janvier, France) were acclimatized for a week before testing. They were housed under controlled environmental conditions (between 21 and 22 °C; 55% humidity, 12 h light/dark cycles, food and water ad libitum). All experiments were approved by the local ethics committees (#18022) and performed according to the European legislation (Directive 2010/63/EU) concerning the protection of animals used for scientific purposes. In vivo and ex vivo fluorescence imaging was realized using the IVIS Spectrum system (Perkin Elmer, Waltham, MA) and a Cy5 filter set (excitation 640 nm; emission 680 nm). Female mice were injected intra-peritoneally with H_2_N-ERα17p-Pra(Cy5)-COOH 2 mg/kg. For in vivo imaging, mice were anesthetized with 2% isoflurane (Aerrane, Baxter, Mississauga, CA) in air/O_2_ (80/20). Acquisitions were realized 15 min and 30 min post-injection. Then, they were sacrificed and uterine horns, ovaries, and skin with abdominal mammary glands were removed to perform ex vivo fluorescence imaging of isolated organs. All images were acquired and analyzed using Living Image 4.7.2 software (PerkinElmer, Waltham, MA). Experiments were realized in the multimodal imaging platform IVIA (Clermont-Ferrand, France).

### 2.9. Docking Studies

In the absence of any experimentally solved structure, the GPER conformation was modeled using the GPCR-I-TASSER server [41], which is expressly dedicated to modeling G protein-coupled receptors. The protein structure was refined in its unliganded form in simulations performed with the GROMACS package [42] and complexed with the ligands using AutoDock Vina for initial prediction of their binding locations [43]. Finally, all-atom molecular dynamics (MDs) simulations in explicit water and in the presence of charge-neutralizing counter-ions were carried out to refine the protein–ligand complexes and to evaluate the effects of the ligand dynamics in GPER binding.

The peptide ERα17p was built from the N-terminal region of the human ERα ligand-binding domain in complex with E_2_ and the E2#23FN3 monobody and deposited in the Protein Data Bank as entry 2OCF [44]. Missing regions including the tetrapeptidic sequence PLMI were reconstructed in silico. Complete conformational freedom was given to all the missing residues of the ligand during the docking procedure and the whole structure was free to rearrange during the MD simulation step.

Molecular docking was carried out with high exhaustiveness of search according to a previously reported procedure [45]. AMBER ff99SB-ILDN [46] and GAFF [47] force fields were used for protein and ligand MD simulations, respectively. After an initial period of equilibration, conformational sampling was performed in the isobaric-isothermal ensemble for 20 ns. Reference values and coupling times used for the barostat and thermostat and other simulation conditions including the modeling of electrostatic and van der Waals forces and treatment of long-range corrections to London dispersion interactions, were as previously reported for other analogous protein–ligand complexes [48,49]. At the end of the MD simulations, the binding modes and the affinity of the ligands were estimated from the structures of the protein–ligand complexes obtained every nanosecond. The binding energy was evaluated by using the AutoDock Vina energy evaluation function [43] in score-only mode.

### 2.10. Statistical Analysis

Statistical analysis was done using ANOVA followed by the Newman–Keuls’ method to determine differences in means. *p* < 0.05 was considered as statistically significant.

## 3. Results

### 3.1. ERα17p Elicits Anti-Proliferative Activity through GPER

**Figure 1:**
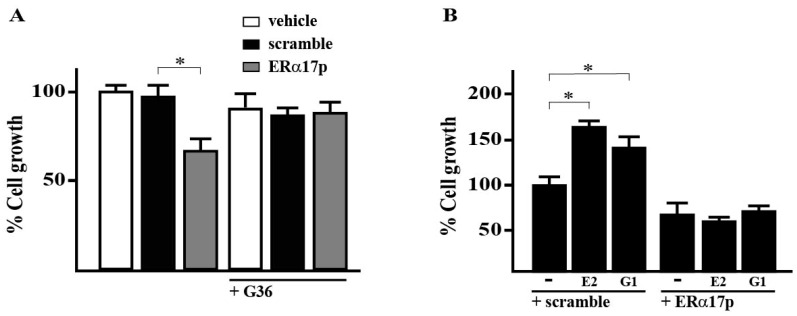
The peptide estrogen receptor α (ERα17p) inhibits the growth of SkBr3 breast cancer cells through the G protein-coupled estrogen receptor (GPER). (**A**) Effects of vehicle, 10 μM scramble peptide (control) and 10 μM ERα17p on SkBr3 cell growth in the presence or absence of the GPER antagonist G-36 (100 nM). (**B**) The proliferation of the SkBr3 breast cancer cells upon treatment with 100 nM E_2_ or 100 nM G-1 is inhibited by 10 μM ERα17p. Cells were treated for three days with the indicated treatments and counted on day four. Proliferation of cells receiving vehicle was set as 100%, upon which cell growth induced by treatments was calculated. Each data point is the average ± SD of three independent experiments performed in triplicate. (∗) indicates *p* < 0.05.

**Figure 2:**
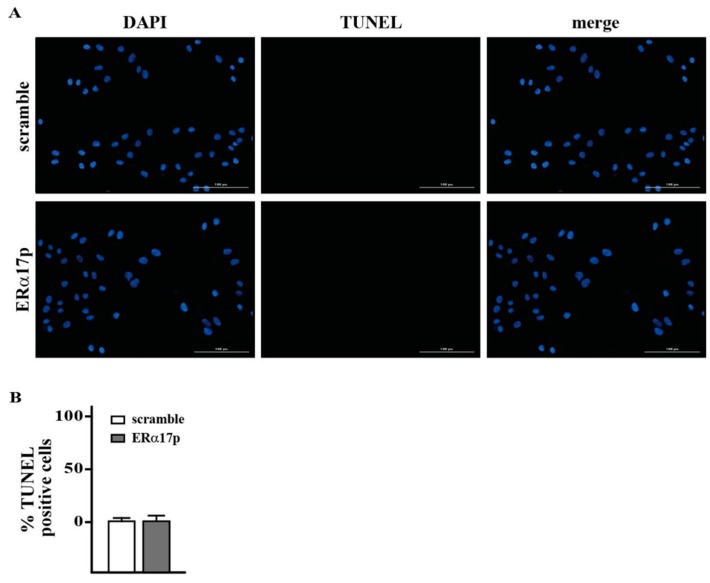
(**A**) Apoptosis detection by TUNEL (TdT-mediated dUTP nick-end-labeling) assay. (**B**) TUNEL staining (green) in SkBr3 cells treated for 72 h with 10 μM scramble peptide (control) and ERα17p. Nuclei were stained by 4′,6-diamidino-2-phenylindole (DAPI) (blue). Magnifications are indicated by horizontal bars (100 μm). Each experiment is representative of 20 random fields observed in each of three independent experiments. Bars graph represents the percentage of TUNEL-positive cells upon treatment versus vehicle. Values are the mean of three independent experiments.

We began our study by evaluating the proliferation of SkBr3 cells in the presence of the peptide ERα17p. After 72 h of treatment with 10 μM ERα17p, we noticed a roughly 25% decrease in the growth of SkBr3 cells (Figure 1A). No effect was observed with the scramble peptide. However, TUNEL assays failed to reveal apoptosis (Figure 2A,B).

Next, we tested the anti-proliferative action of ERα17p in the presence of the GPER antagonist G-36, [50], the GPER agonists G-1 [51] and E_2_ [52], each at a concentration of 100 nM. The antagonist G-36 decreases the anti-proliferative action of ERα17p by ~50% (Figure 1A). The cell growth percentages obtained with G-36, alone, or after a pre-incubation of 72 h with 10 μM ERα17p were identical. We also observed that ERα17p at the same concentration prevents the growth effects induced by 100 nM E_2_ or G-1 (Figure 1B). No effect was observed with the scramble peptide. In any case, no additive effects were observed between ERα17p and the tested GPER ligands. Importantly, a negative cell growth value can be assigned to ERα17p when referred to the GPER in the absence of ligand (normalized reference with the scramble peptide: 100%).

### 3.2. ERα17p and GPER Concomitant Staining at the Cell Membrane

**Figure 3:**
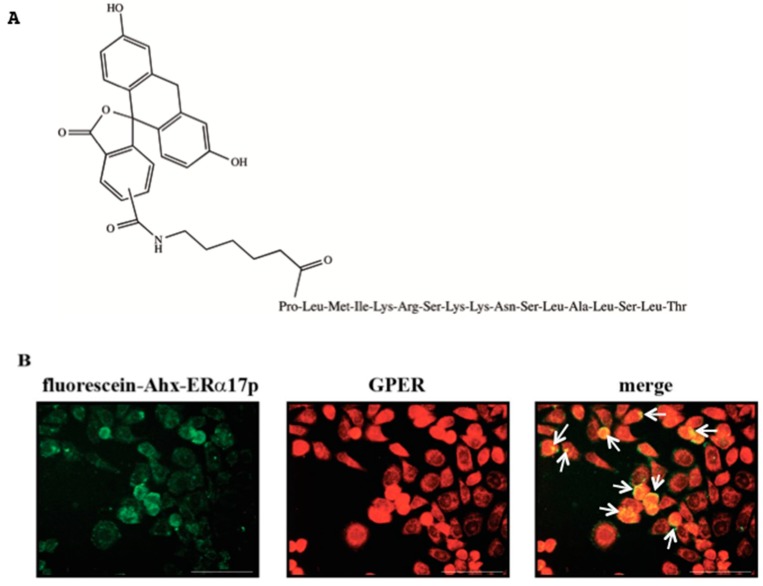
Fluorescence signal of the fluorescein-labeled peptide (carboxyfluorescein-Ahx-ERα17p). (**A**) Structure of the peptide ERα17p labeled at the C-terminus with Ahx (spacer) and carboxyfluorescein. The peptidic part of the molecule is written using the three letter code. (**B**) SkBr3 cells treated for 5 min with the peptide carboxyfluorescein-Ahx-ERα17p (10 μM, green signal, left) or immunostained with the anti-GPER antibody (red signal, middle). The overlay of the peptide carboxyfluorescein-Ahx-ERα17p and GPER signals generates the merge signal (in yellow) visualized in the right panel by white arrows. Each experiment is representative of 20 random fields observed in each of three independent experiments.

The cellular localization of ERα17p was explored by confocal microscopy using an N-terminal carboxyfluorescein-labeled version of the peptide (fluorescein-Ahx-ERα17p, Figure 3A). First, we confirmed that the fluorescein probe had no effect on the activity of the peptide. After 72 h of incubation, the labeled peptide (10 μM) induced 71% of viability (reference: ERα17p: 73%), confirming the absence of probe effect in the biological response. We observed a localization of the peptide at the membrane. The ERα17p fluorescence signal was superimposed with the immunofluorescent stain of the specific GPER antibody TA 55133, after 5 min of incubation (Figure 3B).

### 3.3. Absence of Interaction between ERα17p and Grb2 SH3 Domains

**Figure 4:**
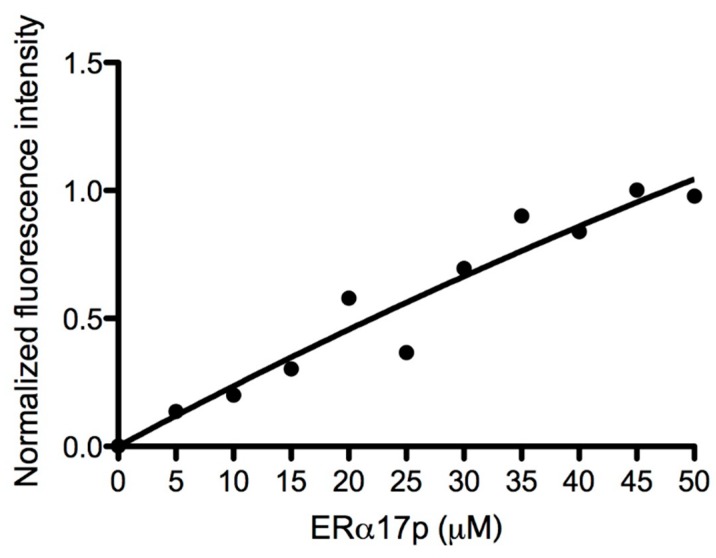
ERα17p/Grb2 SH3 domain interaction study by fluorescence spectroscopy. Fluorescence changes of the sole tryptophan of the Grb-SH3 domain (1 μM in 50 mM Tris buffer adjusted to pH 8) by ERα17p upon successive addition of 5 μL at 10^−3^ M in Tris buffer. Measurements were performed at 18 °C. The λ_exc_ and λ_em_ values were 280 nm and 350 nm, respectively. Experimental data points have been fitted with the software Prism 5.0a.

The GPER works in concert with growth factor receptors, which accept Grb2/Son of sevenless (Sos) as juxtamembrane mediators. We have used fluorescence spectroscopy to explore the interaction between ERα17p and the recombinant heterologous N- and C-terminal Sos-interacting Grb2 SH3 domains (SH3-SH2-SH3). Towards this aim, we have taken advantage of the presence of tryptophan in both Grb2 SH3 domains (Trp-36 and Trp-194 in the N- and C-terminal Grb2 SH3 domains, respectively) prone to fluorescence quenching under ligand association [53]. Fluorescence-based titration assay failed to reveal an interaction between Grb2 SH3 domains and the peptide ERα17p (Figure 4).

### 3.4. ERα17p Downregulates GPER in a Proteasome-Dependent Manner and Decreases the Activation of EGFR and ERK1/2 as well as the Level of c-fos

**Figure 5:**
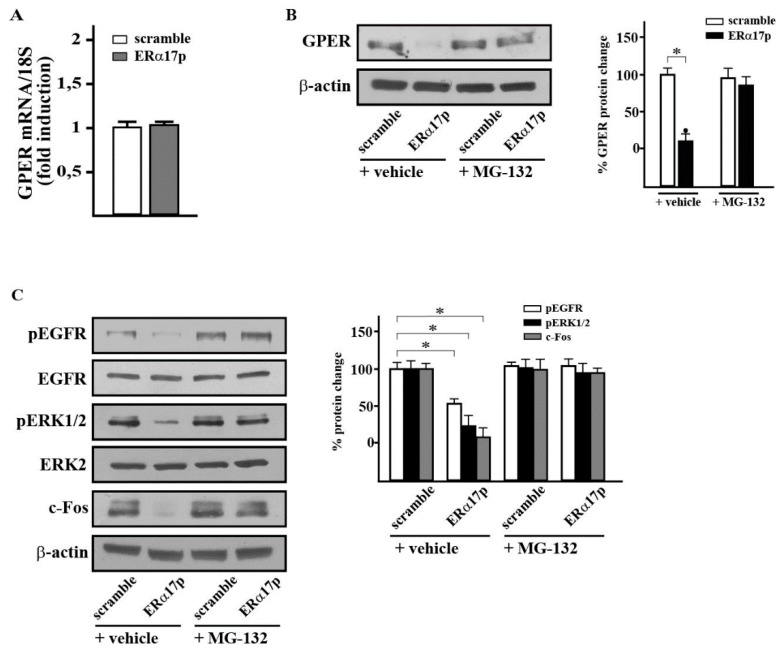
ERα17p downregulates proteins involved in GPER signaling in a proteasome-dependent manner. (**A**) The mRNA expression of GPER was evaluated by real-time PCR in SkBr3 cells treated for 8 h with either 10 μM scramble peptide (control) or 10 μM ERα17p. Results from three independent experiments, each in triplicate are normalized to actin and are shown as fold changes of mRNA expression induced by ERα17p with respect to cells treated with control scramble peptide (control). (**B**) Evaluation of the GPER protein level in SkBr3 cancer cells treated for 8 h with 10 μM scramble peptide (control) and 10 μM ERα17p, in the presence or in the absence of the proteasome inhibitor MG-132. Side panel shows densitometric analysis of the blot normalized to β-actin, which was used as a loading control. (**C**) Evaluation of pEGFR (phosphorylation of epidermal growth factor receptor), pERK1/2 (phosphorylation of extracellular signal-regulated kinase), and c-fos protein levels in SkBr3 cells treated for 8 h with 10 μM scramble peptide (control) and 10 μM ERα17p, in the presence or in the absence of the proteasome inhibitor MG-132. Side panel shows densitometric analysis of the blots normalized to EGFR, ERK2, and β-actin, which were used as loading controls for pEGFR, pERK1/2, and c-fos, respectively. Data are representative of at least two independent experiments. (∗) indicates *p* < 0.05.

At a concentration of 10 μM and after 8 h of treatment, the peptide ERα17p drastically lowers the levels of GPER. It is noteworthy that both ERα17p and scramble peptide (10 μM) failed to decrease the level of GPER mRNA after 8 h of incubation (Figure 5A). Importantly, the proteasome inhibitor MG-132 prevents this decrease (Figure 5B). No effect was observed with the scramble peptide. ERα17p also abolishes the phosphorylation of EGFR (i.e., pEGFR) and ERK1/2 (i.e., pERK1/2), and decreases c-fos, as shown in Figure 5C (control: scramble peptide). 

### 3.5. ERα17p Diffuses Easily in Female Mice to Stain Mammary Glands

**Figure 6:**
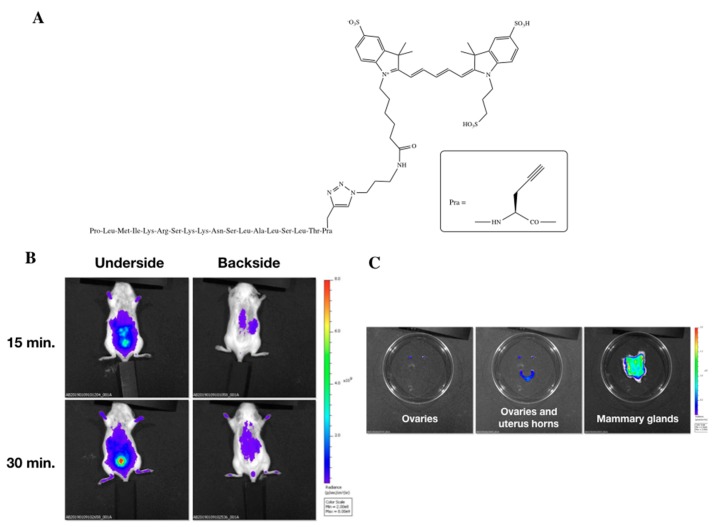
Distribution in mice of the peptide ERα17p at a dose of 2 mg/kg. (**A**) Structure of the Cy5-labeled peptide. (**B**) Pharmacokinetics of the Cy5-labeled peptide after 15 min and 30 min (underside and backside views). (**C**) Distribution of the peptide after 30 min in the ovaries (left), in the ovaries and uterus horns (middle), and in the mammary glands (right).

By using a cy5-labeled version of the peptide (H_2_N-ERα17p-Pra(Cy5)-COOH, Figure 6A), a kinetic approach devoted to its distribution in mice shows that it localizes in the liver and bladder, when intraperitoneally injected at a dose of 2 mg/kg, after 15 min (Figure 6B). After 30 min, which corresponds to the incubation time for which a downregulation of GPER, pEGFR, pERK1/2, and c-fos is observed, it was almost exclusively found in the bladder (Figure 6B). After 30 min, we observed a moderate staining of the ovaries and of the uterus horn (Figure 6C). The labeling was even more impressive in the ventral skin, where the abdominal mammary glands were strongly stained (Figure 6C).

### 3.6. The PLMI Motif of ERα17p Supports the Anti-Proliferative Action of the Entire Peptide

**Figure 7:**
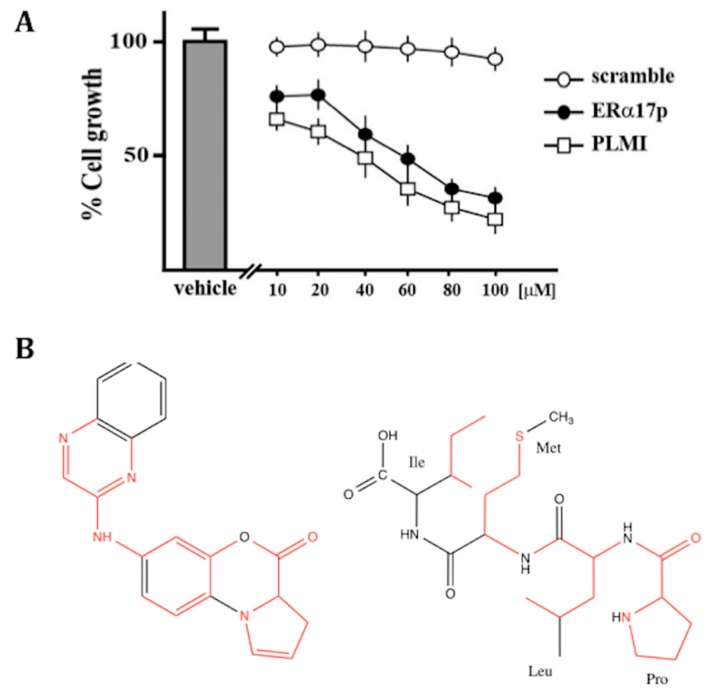
(**A**) The tetrapeptidic sequence PLMI inhibits the growth of breast cancer cells. SkBr3 cells were treated for 72 h with increasing concentrations of the scramble peptide (control), the ERα17p or the PLMI tetrapeptide. Cell viability is expressed as the percentage of cells upon exposure with ERα17p or PLMI with respect to cells treated with the scramble peptide (control). Values are mean ± SD of three independent experiments performed in triplicate. (**B**) Structural analogies (in red) between the GPR30 antagonist PBX1 (left) and the ERα17p-derived peptide motif PLMI (right).

We have evaluated the viability of SkBr3 cells in the presence of the N-terminal peptide fragment PLMI, over 72 h and at concentrations ranging from 10 to 100 μM. Remarkably, the tetrapeptide PLMI and the full-length peptide show comparable dose-dependent anti-proliferative effects (Figure 7A), strongly implying that the N-terminus of ERα17p is the driving force of action of the whole peptide. The scramble peptide, which was used as a control, was inactive.

### 3.7. Docking and MD Studies of the PLMI Motif in the GPER

**Figure 8:**
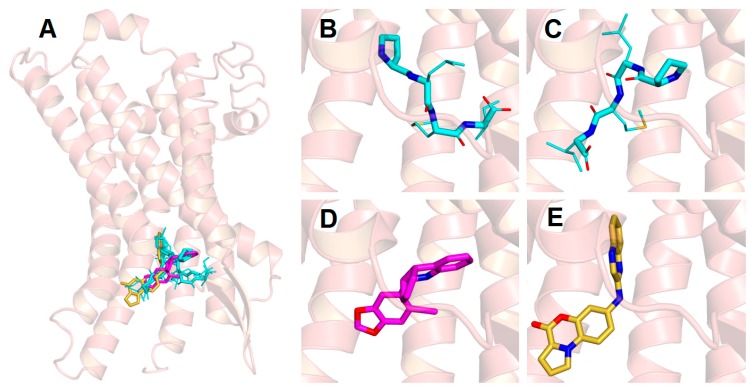
Docking and molecular dynamics (MDs) of ligands/GPER complexes. The GPER is shown as semi-transparent (ribbon), with the disordered region 1–50 omitted. Hydrogen atoms are omitted in all panels. Side chains are represented as smaller sticks compared to the backbone, with the exception of the ring of the proline that corresponds to the N-terminus. The oxygen and nitrogen atoms of the ligands are specified in red and blue, respectively. (**A**) Superimposition on the GPER model of the four most favorable docking structures of the tetrapeptide PLMI (cyan) and of two known GPER ligands (i.e., G-15 (magenta) and PBX1 (yellow)). (**B**, **C**) Details of the two main simulated binding modes of PLMI, with the N-terminus pointing towards the core of the GPER and with the side chain of the proline and the leucine 2 occupying alternate positions. (**D**, **E**) Binding modes of the selected compounds G-15 and PBX1, respectively.

The PLMI motif shares some structural similarities with the putative GPER antagonist 7-(quinoxalin-2-ylamino)-4*H*-benzo[b]pyrrolo [1,2-*d*][1,4]oxazin-7-one (PBX1) [36], as shown in Figure 8B. A blind search performed in a volume including the whole protein surface converged towards an interaction of the PLMI motif in the extracellular domain of the GPER and more particularly within the same cavity as other ligands, including PBX1 (Figure 8A). Predicted binding score values were found to be ~–6.5 kcal/mol (Table 1). The best structures fit with an alignment “head-first”, where the N- and C-termini point towards the protein core and the solvent-exposed region, respectively. Accordingly, the proline and the leucine at the position 2 are deeply inserted in the protein with the two hydrophobic residues able to alternate positions (Figure 8B,C).

Although the initial docked structures had similar binding scores, those structures numbered 3 and 4 showed, even after MD calculations, an energy >–4.3 kcal/mol, which corresponds to a Kd >10^−3^ M (Table 1). The first two structures (N. 1 and 2, Table 1) showed more favorable binding energy (~−5.7 kcal/mol). The ligands PBX1 [36] and G-15 [54], which were used as references, were accommodated within the same protein site with energy values of −8.4 and −7.8 kcal/mol, respectively (Figure 8D, E). The mean values obtained in MD were systematically lower than those values predicted by molecular docking but were still consistent in predicting the association.

**Figure 9:**
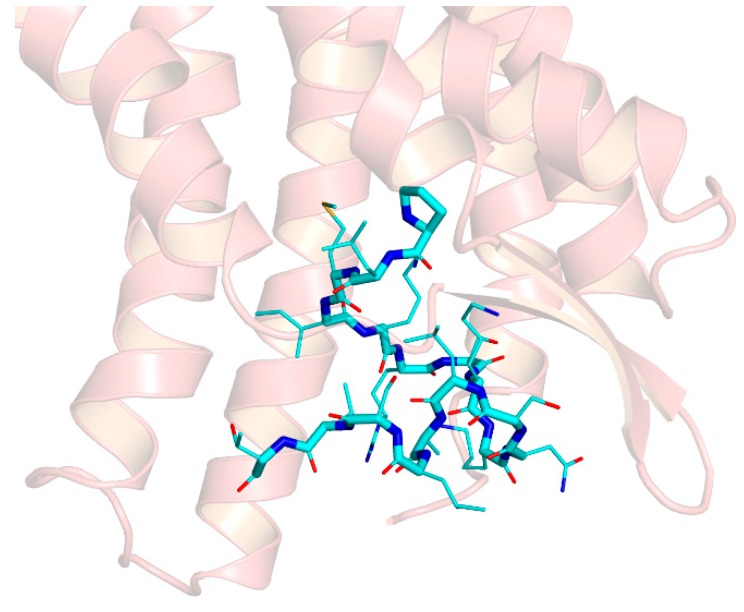
Model of binding of the peptide ERα17p (cyan) to the extracellular region of GPER. The GPER is shown as semi-transparent (ribbon), with the disordered region 1–50 omitted. Hydrogen atoms are omitted in all panels. Side chains are represented as smaller sticks compared to the main chain, with the exception of the ring of the N-terminal proline. The oxygen and nitrogen atoms of the ligands are specified in red and blue, respectively. The N-terminus of the peptide is inserted in the protein core, in a configuration with the first proline residues slightly more plunged within the protein core, whereas the C-terminus is at the entrance of the protein cavity.

As a control, we simulated the binding of the parent peptide ERα17p to the GPER. As shown in Figure 9, ERα17p, as with the PLMI motif, was oriented “head-first” in the same binding site, with the N-terminal region engulfed within the protein core. As in the case of the tetrapeptide, the proline and leucine residues that constitute the first two amino-acid residues could swap their position, determining for the N-terminal group two distinct binding configurations that interconverted during the simulated dynamics. The KRSKKNSLALSLT region of the full-length peptide was compacted at the entrance of the protein cavity. Strikingly, the binding energy was –7.2 kcal/mol, suggesting a Kd value in the low micromolar range.

## 4. Discussion

To distinguish the contribution of ERα from that of GPER, we have turned to the ERα-/GPER+ SkBr3 breast cancer cell line as it is classically used to explore GPER functionality [55,56]. Cell growth assays performed in the presence of ERα17p only, reveal an anti-proliferative activity when compared to those obtained in the absence of ligand (reference: 100% for the scramble peptide, Figure 1A,B). Thus, the GPER seems to harbor a constitutive (intrinsic) activity and implies an “inverse agonism” profile for ERα17p [57,58]. The intrinsic activity of the GPCR is well-documented [59,60,61]. In the same context, we have shown that the GPER antagonist G-36 was able to reduce the anti-proliferative potency of ERα17p (Figure 1A). Likewise, ERα17p abrogates the proliferation induced by E_2_ and G-1 (Figure 1B). As cell growth values were systematically <100% (reference: 100% for the scramble peptide) when the peptide was used, we can conclude an “inverse agonism”. As no synergistic effect was observed, an interaction within the same site as G-1 and G-36 seems likely. The “inverse” agonism profile of ERα17p could explain, at least partially, its opposing action in steroid-deprived conditions, where it stimulates breast cancer cell growth [19,62]. Importantly, the absence of biological response from the scramble peptide (reference), tested in the same experimental conditions as ERα17p, confirms that its action is sequence-dependent and not restricted to charge or other non-specific effects.

The ERα17p-induced decrease of the proliferation of the SkBr3 breast cancer cells logically resulting from either apoptosis or necrosis [35]. Terminal deoxynucleotidyl transferase dUTP Nick End Labeling (TUNEL), which is widely used to explore apoptosis, was carried out. As no small DNA fragments, which are a hallmark of apoptosis [63], were detected (Figure 2), a mechanism of action associated with necrosis must be proposed.

Over 30 min and 60 min, and at a peptide concentration of 10 μM, an ERα17p-induced downregulation of the GPER was detected. Such a phenomenon could result either from a genomic or a proteasome-dependent process. Since the level of GPER mRNA is not affected by the peptide (Figure 5C), a proteasome-dependent mechanism is likely. As the proteasome inhibitor MG-132 is capable of abrogating the GPER level reduction (Figure 5A), a post-translational mechanism is likely. As such, the activation of EGFR and of ERK2 (i.e., pEGFR and pERK2, respectively) as well as of the level of c-fos, were drastically decreased (Figure 5B). Hence, a desensitizing process of GPER should be evoked to explain the mechanism of action of ERα17p.

A crosstalk between GPER and growth factor receptors including EGFR has been demonstrated [64,65,66]. Activated growth factor receptors interact with the juxtamembrane adaptor protein Grb2, which, in turn, binds through its SH3 domains to the 1149–1158 carboxyl terminal polyproline II (PPII) region of Sos (Son of sevenless, sequence: V^1149^PPPVPPRRR^1158^) prior to the activation of the Shc/Ras/Raf/MEK/ERK/Elk-1/c-fos/c-jun transduction cascade [67,68,69]. Thus, we were interested in exploring a potential interaction between the peptide and the Grb2 SH3 domains. We were all the more motivated by this assay given that like the 1149–1158 SH3-interacting region of Sos, the ERα 295–311 sequence is partially structured in PPII [27]. As such, ERα17p could act as a Sos competitor with respect to Grb2. Fluorescence-based titration assays, which were previously described [53], failed to show an association of the peptide with Grb2, giving weight to a direct GPER-mediated mechanism (Figure 4).

In previous studies, we have shown that the peptide ERα17p was able to associate with both artificial anionic [24] and natural [35] membranes. On the basis of these preliminary results we wished to determine if the peptide localized at the membrane of ERα−/GPER+ SkBr3. We have thus used a fluorescein-labeled version of the peptide, where an Ahx (aminohexanoic acid, Figure 3A) was introduced between the fluorescent probe and the peptide to avoid any steric interference that would compromise protein–ligand interactions and, thereby, biological response. The fluorescent peptide was active with the same potency as the parent peptide, validating our approach. Localization of the peptide at the membrane was indeed observed, confirming our previous studies (Figure 3B) [35]. Such results could be related to its weak ability to internalize in cells [24,37,70]. Importantly, the fluorescence signal of the labeled peptide was shown to co-localize with the GPER, as highlighted by a concomitant immunofluorescence stain using the specific GPER antibody TA 55,133 (Figure 3B). Hence, ERα17p membrane targeting may corroborate with a direct association of the peptide within the extracellular GPER ligand-binding site. The full-length 66 kDa ERα and its 36 kDa-truncated isoform, which both share the 295–311 sequence, associate physically with GPER [71,72,73].

A comparison of the structure of the ERα17p with a panel of putative GPER ligands [74] highlighted some structural analogies between the PLMI motif and the N-(4-oxo-4*H*-benzo[b]pyrrolo[1,2-*d*][1,4]oxazin-7-yl)benzamide (PBX1) GPER antagonist (Figure 7B) [36]. Thus, an interaction of the PLMI motif with the extracellular domain of the GPER, where other ligands (including PBX1) bind, is likely. Given these observations, we have used docking and molecular dynamics (MD) simulations to calculate the four most stable GPER/PLMI complexes. Our previous studies on GPER-ligand complexes [74] were also used as a benchmark to assess the accuracy of the theoretical model here used for the protein structure. A number of GPER ligands (including the agonists E_2_ and G-1 and the antagonist G-15) were preliminarily tested to prove their binding within a common region already identified as the protein-binding site. Recognition of the PLMI motif by the extracellular ligand-binding site of the GPER was predicted in the same protein-binding site (Figure 8A–E). The docking procedure allowed us to estimate moderate Kd values (>1 μM). The fact that the mean MD values were lower than those values predicted by docking calculations emphasizes the importance of considering the dynamics of the whole molecular complex for a more accurate estimation of the binding energy. The standard deviations obtained for the two best structures were close to the energetic differences recorded by simple docking and MD simulations and were consistent with the energetic fluctuations resulting from thermal effects at room temperature (~0.6 kcal/mol). These results suggest that molecular docking captures only a static state of the complex corresponding to single isolated minima in the conformational landscape of the tetrapeptide. In other words, an entropic cost due to a decrease of flexibility under peptide/protein association seems likely. The fact that the tetrapeptide remains bound in the same site with a preserved “head-first” configuration during the calculation period validates our model [75,76]. Thus, we have tested the cell growth potency of the peptide PLMI in SkBr3. Importantly, the same effect as the parent peptide ERα17p was observed, further validating our model. These results suggest that only the part of the peptide deeply penetrating in the receptor is responsible for the action of the whole peptide.

The same calculations with the full-length peptide reveal a similar association with the GPER, with a predicted Kd in the micromolar range. The contribution of the first four N-terminal residues was predominant (–5.1 kcal/mol) as the entire peptide remains attached to the GPER ligand-binding site with the N-terminal proline projected towards the receptor core. Due to a significant energy contribution by ~1–3 kcal/mol depending upon the degree of compaction of the unstructured C-terminal region of the peptide (sequence KRSKKNSLALSLT) in the entrance of the protein cavity (Figure 9), we did not attempt to make an accurate estimate of the binding affinity. This effect might depend on finer detail in the parameterization of the solvent that cannot be easily corrected, as suggested by simulations with different water models obtained from a protocol recently developed for disordered peptides and protein regions [77,78].

In a previous work, we have shown that the peptide ERα17p was able to reduce by ~50% the volume of subscapularis xenografted human breast tumors obtained from ERα−/GPER+ MDA-MB-231 basal B TNBC cells [79] in mice without apparently affecting the liver. Thus, a specificity of the peptide for breast tissue seems likely [35]. In the present study, we were interested in exploring the distribution of the peptide in female organs by using an ERα17p peptide labeled at the C-terminus with the bright far-red fluorescent dye Cy5 (ERα17p-Pra(Cy5), Figure 6A), which is ideal for in vivo distribution studies. We observed that ERα17p diffuses easily with typical organ distribution in mice (Figure 6B) and a modest staining of the ovaries and uterus horns (Figure 6C). Likewise, a strong tropism of the peptide for the mammary glands, where GPER is widely expressed, was confirmed (Figure 6C).

In the present study, we have shown that the anti-proliferative action of the peptide ERα17p was mediated by the heptatransmembrane receptor GPER, with which it interacts. Since ERα17p is responsible for a proteasome-dependent downregulation of the GPER, we have concluded that a GPER desensitization mechanism of action from the peptide is involved. A consequent decrease of the level of pEGFR, pERK1/2 as well as of GPER and c-fos was observed. In female mice, the peptide localizes rapidly in GPER rich tissues such as ovaries, uterus horns, and particularly the mammary glands. The N-terminal PLMI motif, which presents strong structural similarities with the putative GPER antagonist PBX1 was shown to support the anti-proliferative action of the whole peptide by locating within the same site of GPER as other ligands. These observations are consistent with the competitive effects of ERα17p with respect to G-1, G-36, and E_2_. In fact, ERα17p acts as an inverse agonist. As such, the motif PLMI could open new avenues for the synthesis of GPER disruptors, which offer hope, as do other GPCR inhibitors, for the treatment of breast cancer [80]. Our work also raises the question as to whether the 295–311 sequence of ERα could correspond to a recruitment platform with the GPER. We would also like to draw attention to the fact that the PLMI motif is, to the best of our knowledge, the first peptidic GPER ligand identified to date.

## Figures and Tables

**Table 1:** Binding energies (in kcal/mol) of the PLMI motif obtained by molecular docking and MD simulation.

Molecular Docking		MD Simulation	
Structures N.	Score	Average	Standard Deviation
1	−6.5	−5.7	0.7
2	−6.5	−5.6	0.6
3	−6.5	−3.7	0.4
4	−6.3	−4.2	0.5
